# Advanced disk herniation computer aided diagnosis system

**DOI:** 10.1038/s41598-024-58283-5

**Published:** 2024-04-05

**Authors:** Maad Ebrahim, Mohammad Alsmirat, Mahmoud Al-Ayyoub

**Affiliations:** 1 Department of Computer Science and Operations Research (DIRO), University of Montreal, Montreal, QC H3T1J4 Canada; 2 Department of Computer Science, University of Sharjah, Sharjah, United Arab Emirates; 3 Artificial Intelligence Research Center (AIRC), College of Engineering and Information Technology, Ajman University, Ajman, United Arab Emirates; 4 Department of Computer Science, Jordan University of Science and Technology, Ar-Ramtha, Jordan

**Keywords:** Lumber disc, Transfer learning, Data augmentation, Feature concatenation, Medical image classification, Computer aided diagnosis, Computer science, Machine learning, Image processing

## Abstract

Over recent years, researchers and practitioners have encountered massive and continuous improvements in the computational resources available for their use. This allowed the use of resource-hungry Machine learning (ML) algorithms to become feasible and practical. Moreover, several advanced techniques are being used to boost the performance of such algorithms even further, which include various transfer learning techniques, data augmentation, and feature concatenation. Normally, the use of these advanced techniques highly depends on the size and nature of the dataset being used. In the case of fine-grained medical image sets, which have subcategories within the main categories in the image set, there is a need to find the combination of the techniques that work the best on these types of images. In this work, we utilize these advanced techniques to find the best combinations to build a state-of-the-art lumber disc herniation computer-aided diagnosis system. We have evaluated the system extensively and the results show that the diagnosis system achieves an accuracy of 98% when it is compared with human diagnosis.

## Introduction

With the advent of biomedical equipment, medical imaging has played an important role in accelerating the diagnosis, monitoring, and analysis of human parts and diseases. That includes radiology medical imaging, such as X-rays, computed tomography (CT) scans, magnetic resonance imaging (MRI), positron emission tomography (PET), and ultrasound imaging. It also includes microscopic medical imaging, such as light, electron, and ion microscopy. While it is the ultimate responsibility of the physician to make the right decision based on these images, the complexity of the images and the wide variety of diseases to capture makes any help to be given to the physician very valuable. Hence, using machine learning to diagnose these medical imaging paves the way for optimized early detection of various diseases and their treatment^1^.

For many years, scientists and researchers have been intensively working on developing and enhancing Computer-Aided Diagnosis (CAD) systems. Those CAD systems can help speed up human work in diagnosing the images produced by the aforementioned medical equipment. For instance, deep learning and machine learning can quickly and accurately classify video and audio recordings of patient interviews compared to, or even better than, human experts^2^. Also, integrating AI-based activity recognition into smart devices enhances their functionality as CAD systems by leveraging their built-in motion sensors^3^. This provides ubiquitous healthcare and wellness monitoring systems that enable personalized monitoring, timely interventions, and behavioral insights.

Continuous flow of patient monitoring data and large-scale medical imaging datasets must be accessed securely to train/retrain data-hungry machine learning algorithms, which contain highly personal and sensitive information about patients^4^. Computer scientists started developing many machine learning algorithms and tools for these types of images, which use hand-crafted features, such as Local Binary Pattern (LBP)^5,6^ and Scale-Invariant Feature Transform (SIFT)^7,8^. These features can be used to classify the images into different classes using machine learning algorithms, such as support vector machine (SVM)^9^ and Artificial/Deep Neural Networks (ANN/DNN)^10,11^.

Inspired by human vision and brain systems, Convolutional Neural Networks (CNN) made its breakthrough in the field of image classification in 2012^12^. CNN has been obtaining state-of-the-art results in many image classification tasks, including medical image classifications. One of the manifestations of the success of CNN is the large-scale benchmark image dataset called ImageNet^13^. Since 2012, Stanford University has been holding an annual challenge on this dataset^14^, which contains around 1.5M accurately labeled natural images categorized into 1K distinct hierarchical categories. This challenge produced several CNN models over the last years that kept on improving the accuracy of classifying this dataset.

Since the development of CNN, many advanced techniques have been introduced to enhance its calcification capabilities. In this study, we are interested in the following:

- Because CNN models are supposed to mimic human vision and brain systems^15^ by generating generic image features of natural images at different levels of abstraction, researchers have proposed transferring the knowledge learned by some ImageNet models into other image classification tasks, including medical image classification^16^. This technique is called Transfer Learning (TL)^17–19^, and it provides a good starting point to train CNN models on new fine-grained tasks.
- The transferred knowledge of different models can be joined together in what is called ensemble learning^20^ and feature concatenation^21^.
- Moreover, a technique called data augmentation^22,23^ has also played an important role in advancing the performance of CNN models on almost every task.

In a previous study^24^, we have created a Lumber Disc Radiology (LDR) dataset composed of axial MRI scans of human spines that were obtained from King Abdullah University Hospital (KAUH), located in Irbid, Jordan. The scans were initially stored as DICOMDIR files, and a radiologist used a DICOM viewer to select the best slice and diagnose it, after which the images were extracted in JPEG format. As a result of the restricted number of patients willing to participate and the high costs associated with gathering patient images, we were only able to amass a modest collection of usable MRI images given the limited funding and personnel resources available to us. The dataset contains a total of 164 images, each sized at 512 $\times$ 512 pixels, and they are categorized into different classes that are detailed in Table 1. In that previous study^24^, we have built a CNN-based CAD system. Due to many limitations, the highest accuracy that we could achieve is only 91%.

In this paper, we systematically explore the impact of different advanced techniques to achieve state-of-the-art performance on our fine-grained medical MRI imaging dataset, i.e., the Lumber Disc Radiology (LDR). These techniques include various transfer learning techniques using different pre-trained CNN models, data augmentation, and feature concatenation. Through this systematic exploration, we achieved state-of-the-art accuracy on the LDR dataset.

Through this work, we provided the basis for a computer-aided diagnosis system that is designed to assist healthcare professionals, not replace them. It will be used to help and guide human experts, who are essential to validate the results produced by the system. Such systems can provide valuable insights for human clinicians, who will have the final clinical judgment, intuition, and the ability to consider various factors when making a diagnosis.

Compared to human experts, CAD systems are better at identifying early signs of diseases that might be challenging for human clinicians to detect at an early stage, leading to earlier treatment. In addition, their quick processing time helps guide healthcare professionals to make faster and more informed decisions, which is especially crucial in emergency situations. Moreover, such systems do not suffer from fatigue and variations in performance, providing reliable consistent results 24/7.

## Background and literature review

In this section, we are going to provide background information and a literature review of different techniques that include three different transfer learning techniques, feature concatenation and ensemble methods, and data augmentation techniques. We also provide information about the dataset used in this work and the previous work on it. We then speak about the nine pre-trained ImageNet models that are used for fine-tuning and feature extraction. Finally, we give a brief information about the six machine learning classifiers that were used on the extracted features.

### Transfer learning techniques

Fine-tuning^25–27^, feature extraction^17,18^, and random initialization^28^ are the three main techniques that are used for transferring the knowledge learned by ImageNet pre-trained models. Of course, transfer learning techniques are not only meant for ImageNet pre-trained models. However, the diversity and the large amount of images in the ImageNet dataset make it the best dataset for transfer learning using CNN models. Over the past years, researchers assumed that CNN models that perform better on ImageNet should perform better on any other recognition tasks.

A very important research by^28^ investigated the goodness of the knowledge/features learned by ImageNet pre-trained models. The results show that ImageNet features are less general than what was expected. Which makes a given transfer learning technique superior to another given the type of the dataset in hand and the information it contains. Moreover, a given model may be superior to another given the data and the transfer learning technique in use. Following is a brief explanation of each one of the most used transfer learning techniques:

#### Fine-tuning

Fine-tuning is one of the most common transfer learning techniques used by researchers^25–27^. In this technique, the learned parameters of the pre-trained CNN model are fully transferred into the new tasks, and then slowly updated to fit the samples in the new classification task. The only modification to the network architecture will usually be changing the number of neurons in the last fully-connected layer to match the number of classes in the new dataset.

#### Feature extraction

To be able to use the knowledge learned by the ImageNet models using other machine learning classifiers, the features of the images in the new classification task are first extracted using an ImageNet model as fixed feature extractors^17,18^. That produces an output feature vector for each image in the new classification task. Those feature vectors are then used to train other machine learning classifiers, such as SVM, to discriminate the different classes in the new task.

#### Random initialization

Fine-grained image datasets, which have images and classes that are very different from those of ImageNet, usually do not benefit from the knowledge learned by ImageNet models. However, they still value the good architecture of those models to produce better classification accuracies on them. That is when random initialization of the ImageNet models is preferred over ImageNet parameters of those pre-trained models^28^.

### Feature concatenation and ensemble methods

Recently, a work by^21^ has proposed the possibility of concatenating the features extracted from ImageNet pre-trained models to provide richer and more discriminative representation for fine-grained image datasets. Ensemble fine-tuning transfer learning technique has been also proposed by^20^ based on the same assumption. Both of those techniques are supposed to produce significant improvements in many fine-grained image datasets, including medical images.

The work by^21^ achieved the state-of-the-art on two well-known microscopic medical datasets. InceptionV3, ResNet152, and InceptionrsnetV2 pre-trained CNN models were used to extract the features from The 2D-Hela and the PAP-smear datasets. These features are then concatenated to train two fully connected DNN layers to classify the images in each one of those datasets. Their results of feature concatenation show a significant improvement compared to feature extraction of a single CNN pre-trained model on both datasets.

Regarding ensemble fine-tuning, the work by^20^ was also able to achieve state-of-the-art results on three benchmark datasets of different domains. Those datasets are the Yahoo! Shopping Shoe, the UC Merced Land Use, and the Caltech Birds datasets. They used AlexNet, VGG19, and GoogleNet pre-trained models to be fine-tuned either for each two models together, or oven for the three models altogether. Their results showed that ensemble fine-tuning achieves better results than single-network fine-tuning. They also proved that ensemble learning is better than feature extraction and feature concatenation on those three datasets.

It is obvious that different models learn different features, and those features perform differently based on the data of a given problem^28^. That fact supports the ideas of both feature concatenation and ensemble learning to produce better classification accuracies on small and fine-grained images, such as medical images. That increases the potential to systematically investigate the effect of those techniques on different types of medical images. That will save time and effort for researchers and practitioners in their specific medical domain.

This work provides a comprehensive study that evaluates the use of individual techniques and their combinations to provide the best approach to handling medical image datasets. Unlike existing studies, where only the final findings of a single approach are shared, without going through what led to those findings.

### Augmentation techniques

Due to the limited number of images, that is usually encountered in small datasets such as medical image datasets, data augmentation has been proposed. It is used to increase the amount of data, as well as increase the diversity in the data to help CNN models work better. Data Augmentation has shown a massive performance improvement for both small and large datasets^12,29,30^.

Besides increasing the amount of data to train on, the main reason for the success of augmentation techniques is that it teaches the model about invariances in the given data. That is because CNN models can only capture translation invariances in the data^12,31–33^. Based on the dataset to work on, CNN models should be able to capture more invariances to work better. Hence, data augmentation provides the needed invariances using the data rather than the architecture, whether or not the given model can capture them.

Moreover, augmentation is used to balance unbalanced datasets^34,35^. That is done by augmenting the images in the classes with fewer samples more than augmenting the images in the classes with more samples. Balancing the datasets using augmentation techniques has shown a great improvement in the performance of natural images^12^, medical images^23,36–38^ and many other problems.

Augmentation techniques usually perform basic image processing operations, such as translation, rotation, and flipping. Sometimes they involve even more complex operations such as intensity and color transformations or even noise addition. Sometimes, a single operation has to be implemented in different levels for every dataset or every class in a dataset. These augmentation operations can produce results comparable to the state-of-the-art even when used with very simple network architectures^23^.

The need for an automatic way to learn the proper augmentation operations on each dataset has been recently raised. Hence^22^, proposed an automatic tool to find the best set of augmentation operations, such as translation, rotation, flipping, or shearing, that should work better on a given dataset. Although it is time and resource-intensive, their AutoAugment tool has obtained state-of-the-art results on many benchmark datasets. The augmentation operations learned by their tool on a given dataset are transferable to similar problems as stated in their work.

The AutoAugment tool works by searching over a predefined set of augmentation policies for each dataset. Each policy is made up of several sub-policies to choose one of them randomly in every training iteration. Each sub-policy contains two transformation operations as well as the magnitudes of these operations and the probabilities to implement these operations.

### Dataset

This work systematically explores the effect of transfer learning, data augmentation, and feature concatenation techniques on the LDR medical image dataset. Table 1 shows the number of classes in our dataset, the number of samples per class, and the number of samples after data augmentation. Following, we discuss some of the important information, characteristics, and state-of-the-art for the LDR dataset.

**Table 1:** The number of samples per class for LDR dataset with and without data augmentations.

Class Name	Samples	Augmented
Left	10	30
Right	5	15
Central	7	21
Diffuse	35	35
Diffuse+Left	4	12
Diffuse+Right	4	12
Diffuse+Central	6	18
Normal	93	93
Total	164	236

**Figure 1:**
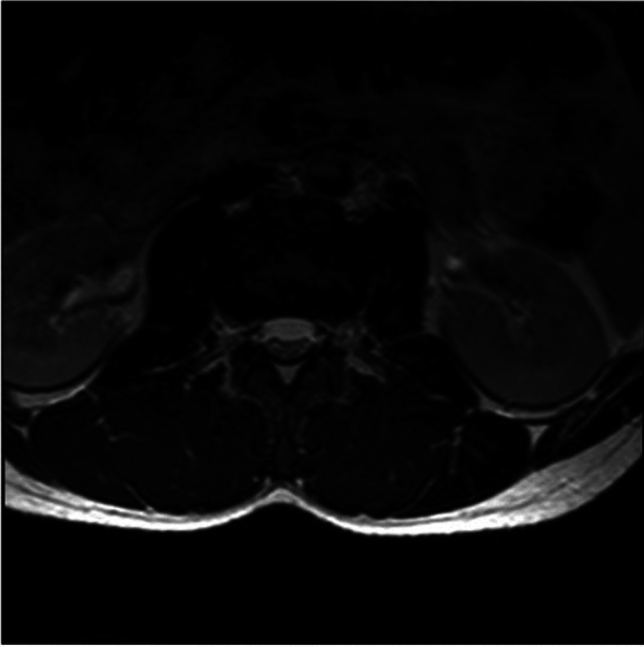
Axial MRI scan for lumbar disk herniation.

Lumbar disc herniation, also known as intervertebral disc herniation, is a common spine disorder causing serious back pain to its patients. X-ray, MRI, and CT scans have all been used to examine the spine for disc problems. But among them, MRI has been widely used and accepted for the diagnosis of lumbar disc herniation abnormality because it shows the internal structures of both bones and soft tissues in the region of interest for diagnoses, as shown in Fig. 1.

The need for CAD systems for lumbar disc herniation disorder has been raised rapidly as it has been the reason for several medical problems^39^. For that reason, a benchmark dataset has been created and used by^8,24,40–43^ axial (top-down) MRI scans instead of the traditional sagittal (side) view scans for disc diagnoses because the axial scan can provide more information about the disc area. Creating this benchmark dataset encouraged researchers to keep finding better approaches for CAD systems of the lumbar disc herniation disorder. Over the last few years, this benchmark has been enhanced by researchers to address some minor issues in the first version of the dataset.

The most recent work in^24^ focused on the use of state-of-the-art techniques, such as CNN, where the feature extraction is automatically performed. Transfer learning was also used in that work to facilitate and speed up the training job using the AlexNet pre-trained model. They achieved an accuracy of 95.65% for the binary classification task. However, they achieved 91.38% for the recognition task, in which the images are classified into eight categories. The recognition accuracy was produced with the help of a slight data augmentation of two rotation operations to increase the number of samples in small categories. This augmented version of the dataset is used in this work, referring to it, in this paper, as “*Disc*” for simplicity.

### Pretrained models

MATLAB^44^ has published several pre-trained CNN models trained on over a million ImageNet^13^ images to classify them into 1000 natural object categories. Those models have often produced state-of-the-art results in the ImageNet Large-Scale Visual Recognition Challenge (ILSVRC)^14^. MATLAB’s Deep Learning Toolbox provides those pre-trained ImageNet models to be used for classification, feature extraction, and fine-tuning using a few lines of code.

**Figure 2:**
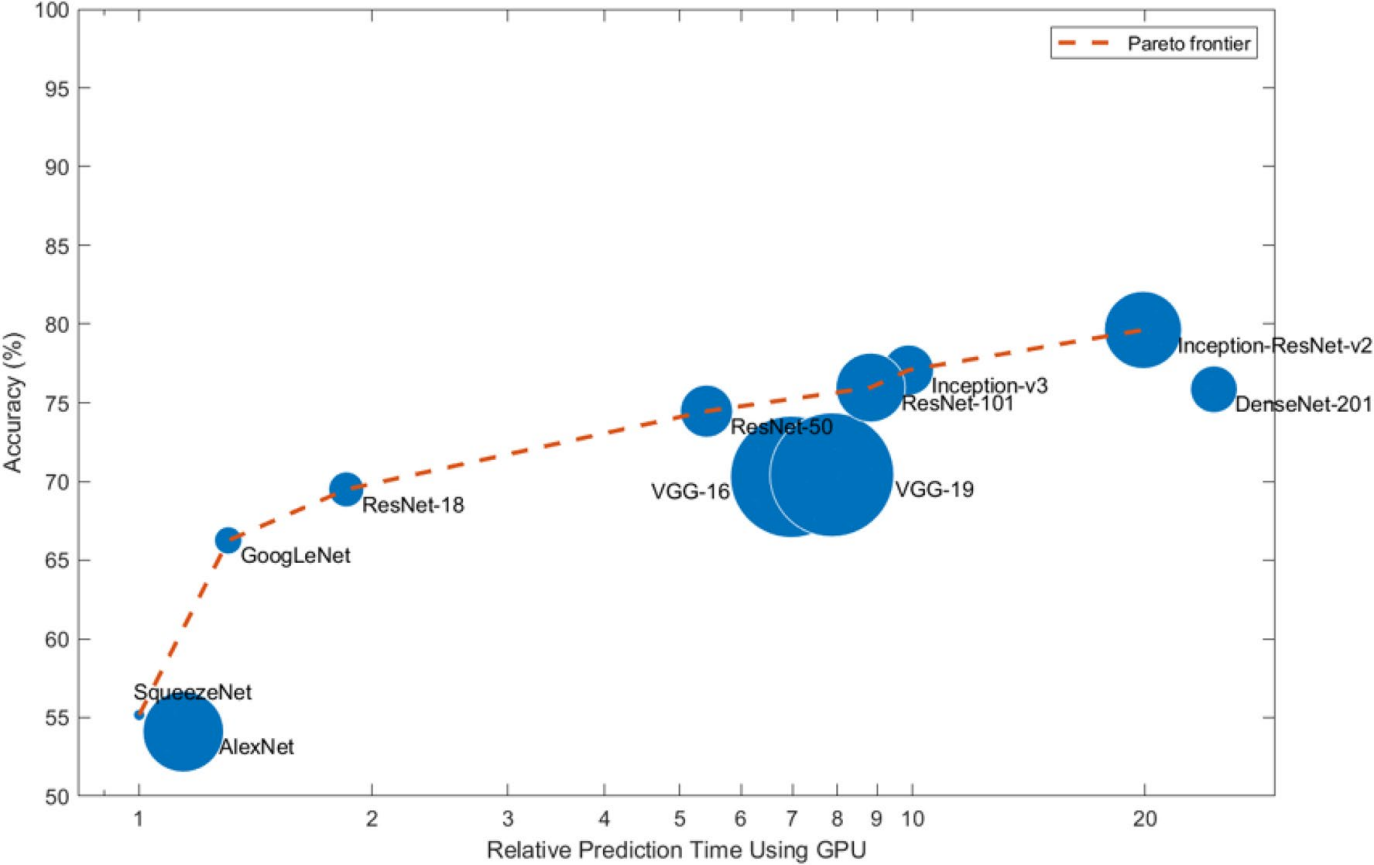
Validation accuracy vs. prediction time for MATLAB ImageNet pre-trained models^52^.

In this work, we selected nine of those pre-trained models to perform feature extraction and fine-tuning on the LDR dataset. Learned from MATLAB documentation, Table 2 shows some of the characteristics of those models. Figure 2 compares the validation top-1 accuracy of each model on ImageNet data with the time required to make a prediction using that model. The results shown in the figure were produced by the MATLAB team using mini-batches of 64 images on a modern GPU (an NVIDIA^®^ TITAN Xp). The prediction time in the figure is relative to the fastest network and the area of the markers represents the size of the network on disk.

**Table 2:** The characteristics of the nine pre-trained ImageNet models used in this work.

Models	Depth	Size	Parameters	Input Size	Top-1	Top-5
AlexNet^12^	8	227	61	227 $\times$ 227	0.4355	0.2049
VGG16^45^	16	515	138	224 $\times$ 224	0.3426	0.1346
VGG19^45^	19	535	144	224 $\times$ 224	0.3411	0.1317
GoogleNet^46^	22	27	7	224 $\times$ 224	0.3227	0.1170
InceptionV3^47^	48	89	23.9	299 $\times$ 299	0.2337	0.0675
ResNet50^48,49^	50	96	25.6	224 $\times$ 224	0.2925	0.1014
ResNet101^48,49^	101	167	44.6	224 $\times$ 224	0.2762	0.0925
InceptionResNetV2^50^	164	209	55.9	299 $\times$ 299	0.2039	0.0522
SqueezeNet^51^	18	4.6	1.24	227 $\times$ 227	0.4288	0.1988

*Depth*, is the largest number of sequential convolutional or fully connected layers on a path from the input layer to the output layer; *Size*, is measured in MB; *Parameters*, are measured in millions; *Input size*, is the image input size of the network in pixels; *Top-1 & Top-5*, are the Top-1 & Top-5 error rates (0 means no error) respectively.

The success of these nine pre-trained models on the ImageNet dataset suggests that they have learned meaningful features from those images, making them good candidates for transfer learning. In addition, these models represent a diverse set of neural network architectures; ranging from relatively simple architectures like AlexNet and SqueezeNet to more complex ones like InceptionV3 and InceptionResNetV2; which enables them to capture different representations from the ImageNet data. This diversity can help us understand the ability of each architecture to generalize to significantly different types of tasks. Using models with different levels of complexity will show which model can perform better than others on fine-grained image recognition tasks, i.e., medical image classification.

### Machine learning classifiers

The extracted features of different pre-trained networks can be used on any classifier to discriminate between the different classes in a dataset. The authors of this work used a deep learning approach and a none-deep learning approach for feature classification. For the deep learning approach, the authors used a multilayer deep neural network (DNN), also called a multi-layer perceptron (MLP) in the literature. This multilayer DNN model is simply made up of multiple fully connected layers to be trained on the extracted features. On the other hand, for the none-deep learning approach, the authors of this work used six different machine learning classifiers, i.e., support vector machine (SVM)^53,54^, Linear SVM^55^, Discriminant Analysis Classification^56,56^, K-Nearest Neighbors (KNN)^57^, Naïve Bayes^58,59^, and Decision Trees^60^.

Since we used the MATLAB implementation of the pre-trained models, we also used the fitCECOC^61^ MATLAB implementation for the six machine learning classifiers with their default parameters. This allows for seamless interoperability to simplify the experimentation workflow. The efficient implementations and the extensive documentation make MATLAB the best choice for researchers and practitioners alike, which allows them to reproduce published results for validation and improvement. While other classifiers exist, these six classifiers cover a broad spectrum of machine learning techniques, from linear models like Linear SVM and Naive Bayes to more complex ones like Decision Trees and KNN. Hence, evaluating these six classifiers is enough to guide future research on specific categories of machine learning classifiers.

## Methodology

In this paper, we propose a systematic exploration of different transfer learning techniques to try to produce state-of-the-art results on the slightly augmented LDR medical image dataset. The nine pre-trained networks mentioned in Table 2 were used for fine-tuning and feature extraction on the images of those datasets. Those features were then used on their own, or concatenated together for every two pre-trained networks to produce double features. The single and double features are then used to train six different machine learning algorithms as well as a multilayer Deep Neural Network.

We used MATLAB version R2018b, and their implementations of the nine pre-trained networks mentioned in Table 2. These networks were used for both fine-tuning and feature extraction of the images in the two datasets. MATLAB was also used to train the six machine learning algorithms mentioned in Fig. 4. For the multilayer DNN, the authors used a TensorFlow DNN-Classifier estimator in Python to be trained on the features extracted from MATLAB. TensorFlow was chosen for DNN over MATLAB because it provides a more robust and powerful DNN implementation that overcomes the MATLAB implementation for DNN.

**Figure 3:**
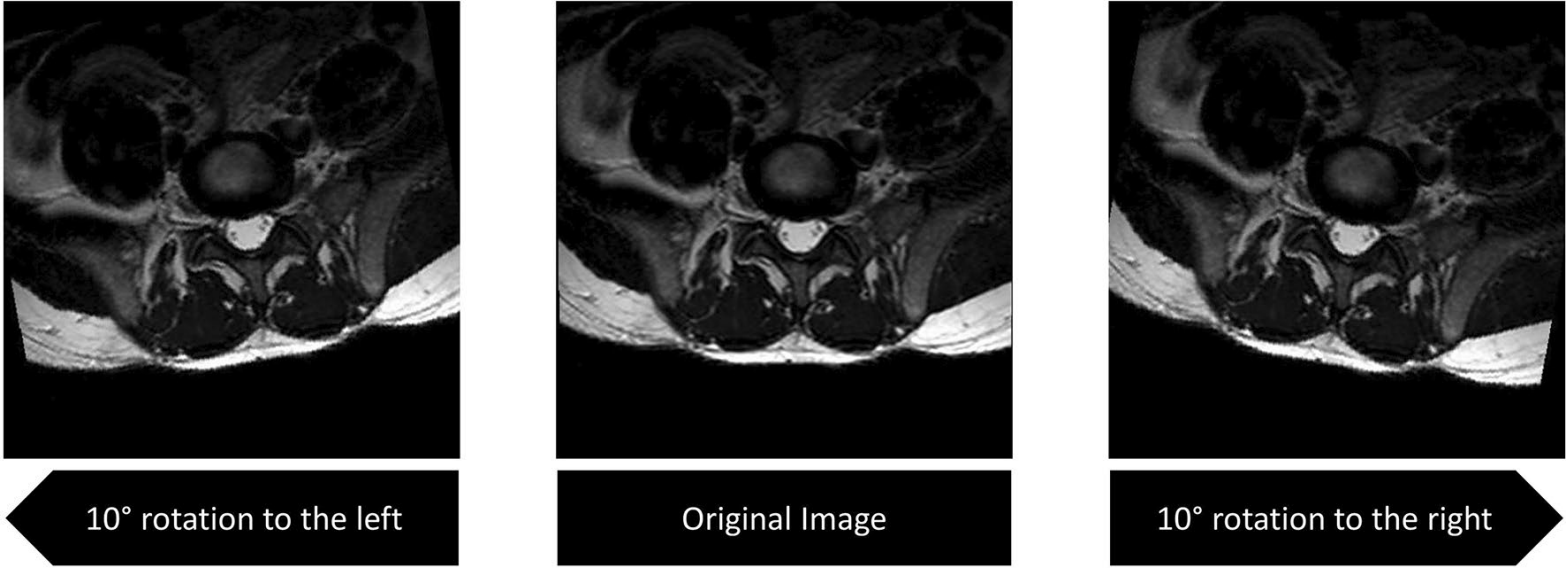
Augmenting the Disc images by two rotation operations.

The LDR dataset is unbalanced, with 236 images categorized into 8 classes, as shown in Table 1. The smallest class contains 12 images, while the largest “normal” class contains 93 images. Each Disc image is a 100 $\times$ 100 colored image cropped from an MRI slide of lumbar disc herniation. The dataset is slightly augmented as in^24^, using just two rotation operations of 10 degrees in both left and right directions, as shown in Fig. 3.

**Figure 4:**
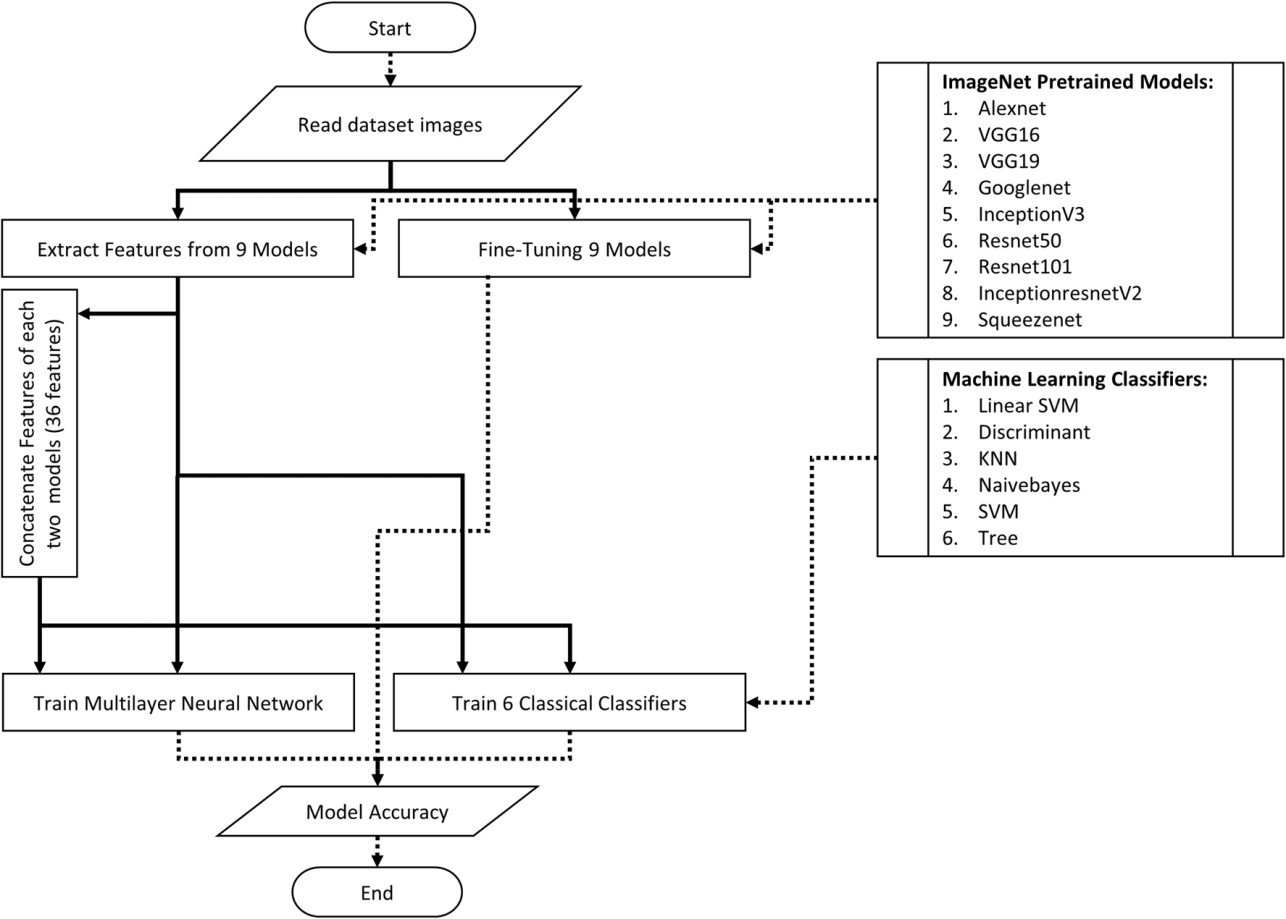
Experimentation flowchart.

Figure 4 shows a workflow of all experiments in this work. The experiments start by reading the images of the LDR dataset. The images are scaled first to match the input of each pre-trained model. They are then shuffled and used for fine-tuning or feature extraction. Finally, The extracted features are used to train a multilayer DNN and the six machine learning classifiers either as single or double features.

### Fine-tuning

The fine-tuning part of the experiments in this work is done without feature concatenation for simplicity and speed. Feature concatenation in the case of fine-tuning is usually referred to as ensemble fine-tuning. Ensemble fine-tuning requires the concatenation of two or more networks at one of their last layers to be trained together. Besides the need to implement the proper cost estimation function on the concatenated networks, it is required to load all concatenated networks in memory to train them as a single network. Hence, implementing ensemble fine-tuning in our work will be extremely hard using our moderate set of devices.

To produce consistent and trusted results, we have averaged the fine-tuning results of ten runs. Very small mini-batch size, i.e. eight images per iteration, has been used in our fine-tuning experiments. That is due to the large size of the used pre-trained networks, and our limited availability of resources. The small mini-batch size enables researchers and practitioners to re-implement any part of these experiments on their own moderate set of devices.

MATLAB augmented image data source has been used to automatically resize the images of the dataset. Resizing the images is required to match the proper image size of the input layer of each pre-trained network. To speed up this preprocessing step, the resizing operations are done using a background execution of four MATLAB parallel workers. The networks were fine-tuned for 40 training epochs. The training set is shuffled for every epoch and trained using the Stochastic Gradient Descent with Momentum (SGDM) optimizer with a $1\textrm{e}{-4}$ constant learning rate.

To be able to retrain those pre-trained networks, we first replace the last learnable layer of the network, which is usually a fully-connected layer. The number of neurons in that layer should match the number of classes in the new task. We also replace the final classification layer, which computes the cross entropy loss for multi-class classification problems of mutually exclusive classes. The replacement of the classification layer is needed to adapt the network to the new class labels in the new data set.

**Figure 5:**
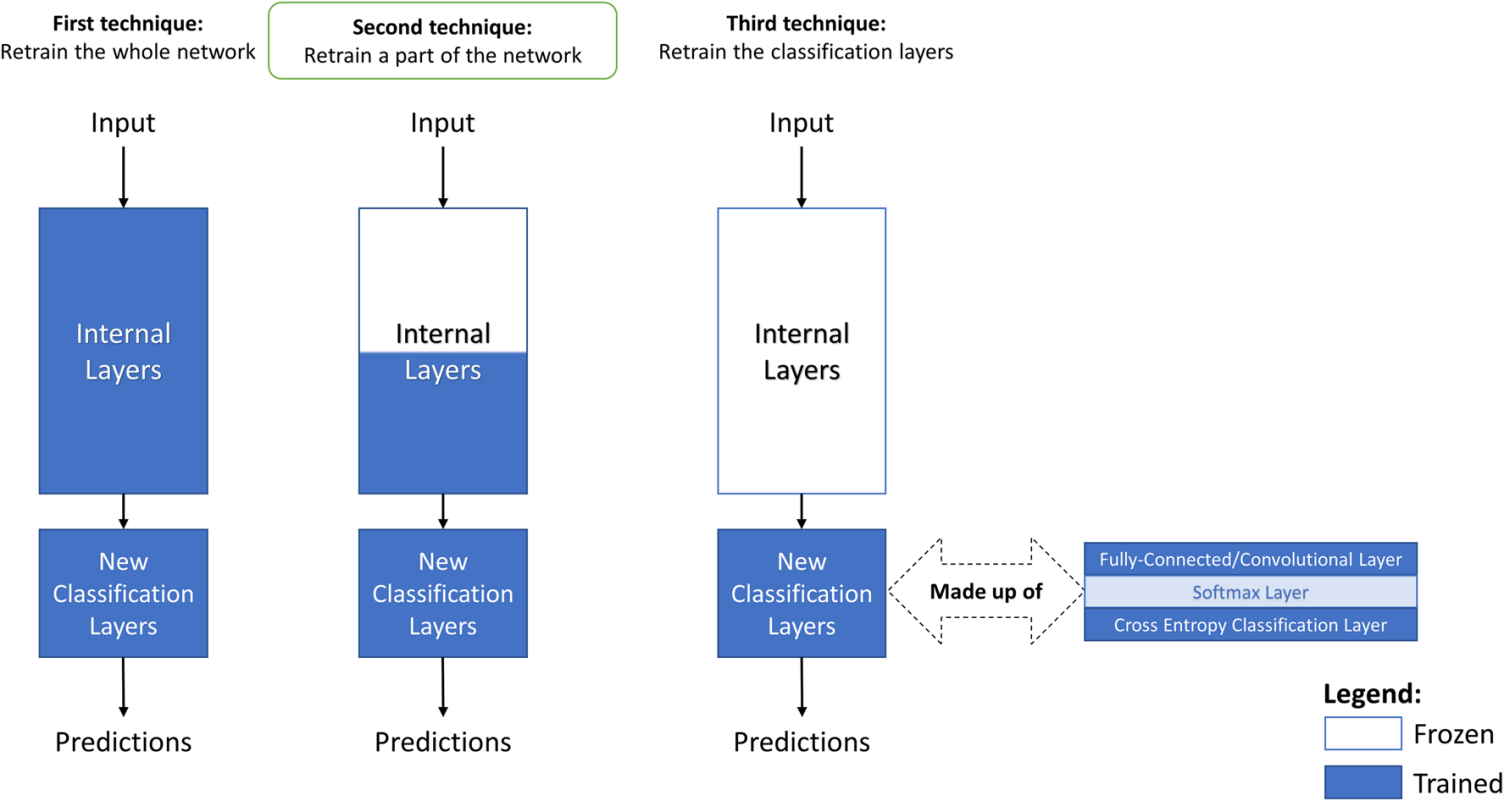
Three well-known fine-tuning techniques.

To learn faster in the newly added layers, we increased the learning rate factors of those layers relative to the learning rates of the original layers. Moreover, we froze the weights of the first 10 layers in the network by setting the learning rates in those layers to zero. Figure 5 shows the three different strategies that are usually used for fine-tuning. As shown in the figure, we implement the second fine-tuning strategy. Freezing the earlier network layers significantly speeds up the training time. It also prevents the overfitting problem that neural networks usually encounter on small datasets.

### Feature extraction and concatenation

Feature extraction is done first to transfer the knowledge of the nine pre-trained networks to the multilayer DNN and the six machine-learning classifiers. For each pre-trained network, we extract the features of each image to create image feature vectors. We used MATLAB augmented image data source again to automatically resize the images to the proper image size of the input layer of each pre-trained network. The training set is pre-shuffled to provide robust data distribution for the machine learning classifiers, and the multilayer DNN to perform better training on the data.

Each image is represented by a feature vector of a length that corresponds to the number of neurons in the feature extraction layer of the pre-trained network used, as shown in Table 3. The table also shows that the feature extraction layer is usually the first fully-connected layer for each pre-trained network. The first fully connected layer is selected to capture the features of the last feature extraction layer in the network.

**Table 3:** The layer name and its length (number of neurons) used for feature extraction of each of the nine pre-trained models in MATLAB.

Model name	Extraction layer type	Layer name	Feature length
AlexNet	The 1st fully-connected	fc6	4096
VGG16	The 1st fully-connected	fc6	4096
VGG19	The 1st fully-connected	fc6	4096
GoogleNet	The only fully-connected	loss3-classifier	1000
InceptionV3	The only fully-connected	predictions	1000
ResNet50	The only fully-connected	fc1000	1000
ResNet101	The only fully-connected	fc1000	1000
InceptionResNetV2	The only fully-connected	predictions	1000
SqueezeNet	The last average pooling	pool10	1000

Some pre-trained models only have a single fully-connected layer for classification. While in the SqueezeNet model, there are no fully-connected layers in its implementation to be used as a learnable layer. The SqueezeNet features are therefore extracted from the last average pooling layer, which is actually the last layer before the softmax and classification layers.

**Figure 6:**
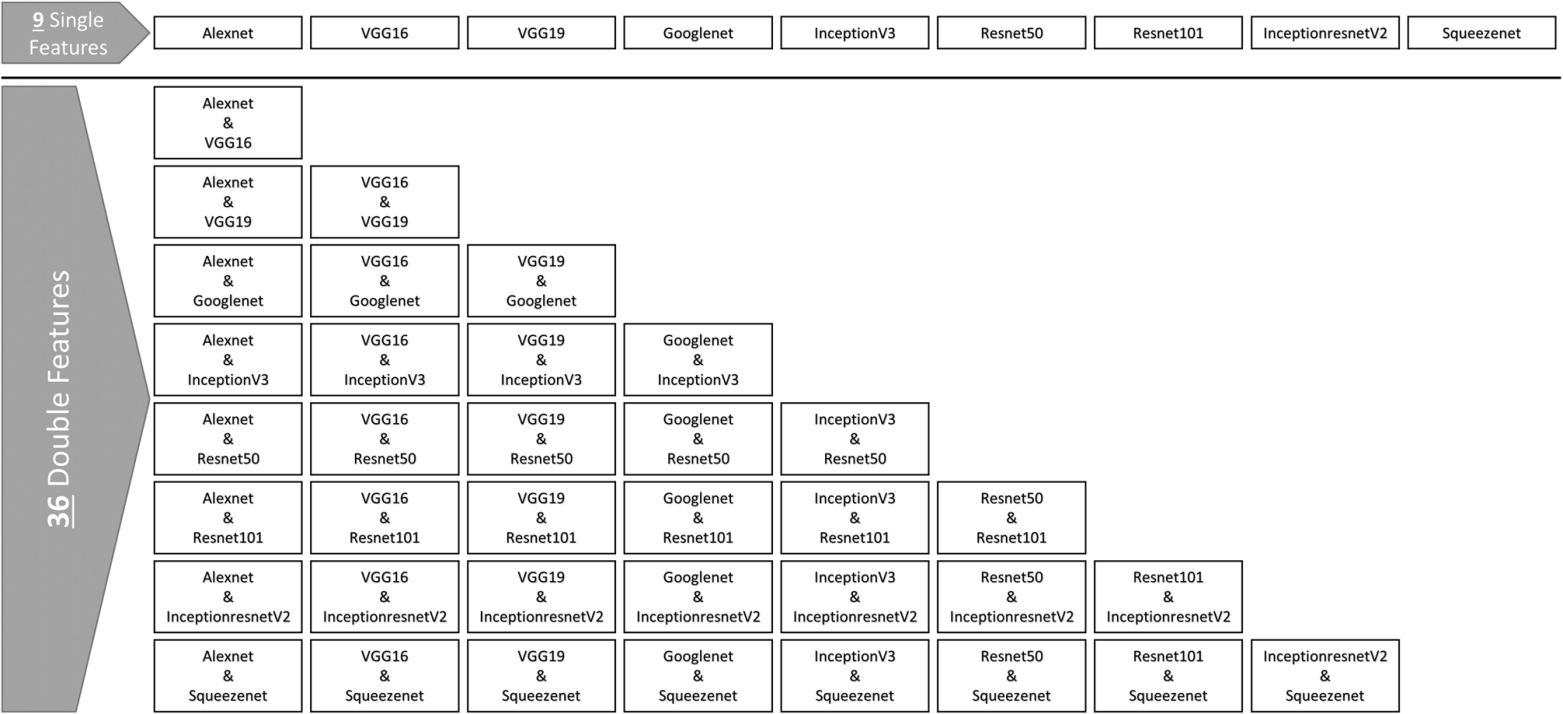
36 double concatenated features from 9 single-features.

The extracted features are directly used as single features to train the multilayer DNN and the six machine-learning classifiers. Furthermore, the features extracted from every two pre-trained models are concatenated together to form larger feature vectors, as can be seen from Fig. 6. The produced double features are of very high dimensionality, especially when AlexNet, VGG16, or VGG19 features are used for concatenation. That is because their single features already suffer from the curse of dimensionality problem, with a feature size of 4096 features (neurons). Concatenating each two of those high dimensional features produces a feature vector of length 8192.

### Multilayer deep neural network (DNN)

In this work, we use the DNN-Classifier estimator of the TensorFlow framework. Both single and double features are used to train the multilayer DNN. We start by creating an input layer with the number of neurons equal to the number of features in the feature vectors. Since the length of each feature vector differs for each pre-trained model, then the number of neurons in the input layer will also differ for both single and double features of different pre-trained models.

Our DNN network is composed of three hidden fully-connected layers of 1000 neurons each. We have used Batch normalization for each hidden layer in the network. We have also used a 50% dropout factor, and tanh activation function for each hidden layer. Adagrad optimizer is used with $1\textrm{e}{-3}$ constant learning rate factor. Weighted-mean loss reduction is used to reduce the loss over a single batch for every training iteration. A mini-batch size of 128 feature vectors was used for every training epoch. The training was performed for 300 training epochs. The training input data is shuffled for every epoch during the training time.

### Machine learning classifiers (ML)

We again used both single and double features to train the six different machine learning classifiers mentioned in Fig. 4. For each one of the six classifiers, we use four MATLAB parallel workers to accelerate the code execution on our moderate set of devices. FitCECOC was used to fit the *Linear SVM*, *Discriminant*, *KNN*, *Naïve Bayes*, *SVM*, and *Tree* multi-class classifiers.

Observations, i.e. samples, in the data correspond to rows for all classifiers except for the linear SVM learner, where observations correspond to columns in the data. As per MATLAB documentation, this data representation provides a significant reduction in the optimization and execution time. we fit the FitCECOC learners using a one-versus-all coding scheme for multi-class classification. And for all those classifiers, we used them with their default options and parameters, to avoid the long process of hyperparameter optimization and tuning for each one of them.

## Results and discussions

With such a large number of experiments, and consequently, a large number of results, using a single metric is better to focus on one clear measure of performance when interpreting the results. This makes it easier to compare different solutions and quickly identify the best-performing approach without getting lost in a sea of numbers. Accuracy is a very good indicator of overall model performance, which can give a clear picture of how well the model is doing. Accuracy is enough by itself when the dataset has a roughly equal number of samples for each class. Hence, performing data augmentation to minimize sample variation in each category makes accuracy the best performance metric to choose from.

Hence, we use the accuracy measure for all reported results in our experiments on a scale between 0 and 1, where 1 represents the best possible accuracy with no errors. The reported accuracy values are rounded to only two decimal places in the given figures and tables, to make it easily readable. The reported accuracy measure is calculated on the validation set of the dataset, and calculated as shown in the following equation:

$$\begin{aligned} Accuracy=\frac{Correct \, Predictions}{Total \, Number \, of \, Samples} \end{aligned}$$

Due to the large amount of results in this work, we represent the results per dataset for easier discussion. For each dataset, we discuss the results of single and double features separately for each transfer learning technique. Fine-tuning results are represented first as they were implemented using only single features. Moreover, fine-tuning works directly on the images of each dataset to tune the parameters of the pre-trained networks, instead of using them as fixed feature extractors.

The results are shown in tables for the DNN and machine learning classifiers to make them more readable. However, fine-tuning results are represented in figures with a small data table below each figure. The data table shows the maximum, minimum, and average accuracy over all the runs for every pre-trained model. We later provide a detailed discussion of the provided results. We also discuss the effect of data augmentation and feature concatenation on the used classifiers.

It was quite fast to produce the results for fine-tuning, DNN, and ML classifiers. That is due to the small size of the dataset, although it has been slightly increased by a couple of rotation operations for some of its classes. The small size of the dataset comes at the cost of producing inconsistent results as will be discussed later.

The dataset is split into 75% and 25% for training and testing, respectively. Which is the same way that has been implemented in the work by^24^. A random split of the dataset into training and testing sets is the most used approach in the literature. In addition to being a common approach, we chose data splitting over more computationally intensive methods like K-Fold cross-validation due to the large number of experiments and the limited availability of computational resources to perform those experiments.

It is good to note that the augmentation done by^24^ on the LDR dataset significantly helped in achieving the best results in their work. It also helped boost the accuracy of the results reported in this work. However, more augmentation operations should also be investigated as it might help boost the accuracy even further.

### Fine-tuning

**Figure 7:**
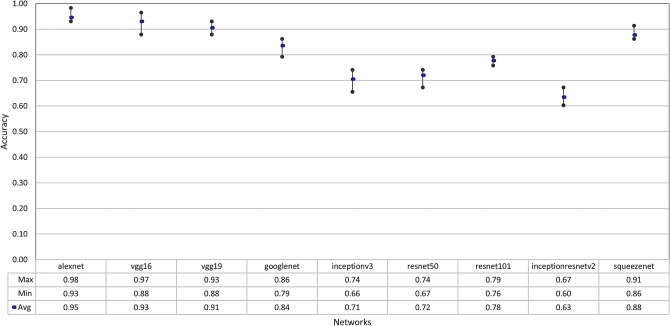
Fine-tuning results over 10 runs.

Starting with the fine-tuning results of the LDR dataset images, Fig. 7 shows the maximum, minimum, and average accuracy values over 10 fine-tuning training runs on each of the nine pre-trained networks. Each network was fine-tuned for 40 training epochs in every run, which did not take too much time due to the small size of the dataset.

We can notice that the average accuracy values significantly vary for each pre-trained network. AlexNet produced the best average accuracy on the LDR dataset with a maximum accuracy of 98%. On the other hand, InceptionResNetV2, which outperforms all the eight pre-trained models on the ImageNet dataset, produced the worst average accuracy on this dataset with a maximum accuracy of 67%.

The results show that Inception and ResNets architectures are not performing well on the LDR dataset. And these results support the findings of^28^, that ImageNet features are less general than what was previously suggested. This means that the parameters learned by a good model on the ImageNet dataset are not necessarily a good starting point to train a fine-grained medical image dataset of bones and tissues, such as the images in the LDR dataset.

### Single-features

Linear SVM classifier is significantly faster than all other classification methods. The accuracies for the LDR single features using DNN and the six machine learning classifiers are shown in Table 4. The best feature extractor differs based on the classification method used on the extracted features. However, the ResNet50 feature extractor produced the best classification accuracies on DNN, Linear, and Tree classifiers.

**Table 4:** Accuracies of DNN and ML classifiers using *single-features*.

Model	DNN	Linear	Discriminant	KNN	Naïve Bayes	SVM	Tree
AlexNet	0.47	0.74	0.78	**0.88**	0.64	***0.95***	0.45
VGG16	0.76	**0.81**	0.78	0.86	0.66	0.91	0.45
VGG19	0.74	**0.81**	**0.83**	0.86	0.66	0.93	0.43
GoogleNet	0.79	0.66	0.59	0.78	0.50	0.74	0.41
InceptionV3	0.81	0.76	0.66	0.76	**0.69**	0.81	0.41
ResNet50	**0.88**	**0.81**	0.64	0.78	0.60	0.88	**0.59**
ResNet101	0.81	0.76	0.72	0.81	0.62	0.86	0.40
InceptionResNetV2	0.83	0.72	0.69	0.81	0.59	0.74	0.52
SqueezeNet	0.45	0.59	0.76	0.72	0.47	0.84	0.55

The highest accuracy for each classifier is highlighted in bold font.

The highest overall accuracy is highlighted in bolditalic font.

AlexNet produced the best classification accuracy, i.e. 95%, for the single-feature experiments using the SVM classifier. That accuracy is not better than the best fine-tuning accuracy of 98% using an AlexNet pre-trained network. However, SVM was able to produce pretty good results for each one of the nine pre-trained networks. That makes SVM a good choice for practitioners when working with radiology images using the features extracted from most of the given pre-trained models.

It should be noticed though, that the worst feature extractors for the SVM classifier are the Inception architectures, i.e. GoogleNet, InceptionV3, and InceptionResNetV2. This shows the bad effect of Inception models on this type of radiology medical images. On the other hand, The worst classifiers in our single-feature experiments are Tree and Naïve Bayes classifiers with 59% and 69% best accuracies, respectively.

### Double-features

Again, the linear SVM classifier is significantly faster than all other classification methods. Table 5 shows the accuracies of DNN and the six machine learning classifiers on the double features. For these sets of experiments, there are two feature extractors for each classifier instead of one.

**Table 5:** Accuracies of DNN and ML classifiers using *double-features*.

Model	DNN	Linear	Dis.	KNN	Naïve Bayes	SVM	Tree
AlexNet & VGG16	0.45	0.79	0.79	0.90	0.69	0.95	0.45
AlexNet & VGG19	0.41	0.78	0.74	**0.93**	0.62	0.97	0.45
AlexNet & GoogleNet	0.45	0.71	0.81	0.88	0.67	0.95	0.41
AlexNet & InceptionV3	0.48	0.74	0.79	0.88	0.66	0.95	0.41
AlexNet & ResNet50	0.50	0.84	0.81	0.88	0.66	0.95	0.55
AlexNet & ResNet101	0.55	0.69	0.81	0.88	0.67	0.93	0.43
AlexNet & InceptionResNetV2	0.53	0.78	0.74	0.88	0.66	0.95	0.43
AlexNet & SqueezeNet	0.43	0.60	0.79	0.88	0.66	0.95	0.47
VGG16 & VGG19	0.59	0.81	0.78	0.86	0.62	0.97	0.45
VGG16 & GoogleNet	0.72	0.79	0.76	0.88	0.66	0.91	0.45
VGG16 & InceptionV3	0.66	0.81	0.79	0.86	0.69	0.93	0.45
VGG16 & ResNet50	0.71	0.81	0.81	0.86	0.69	0.93	**0.59**
VGG16 & ResNet101	0.76	**0.86**	0.84	0.86	0.67	0.93	0.52
VGG16 & InceptionResNetV2	0.72	0.79	0.84	0.86	0.66	0.91	0.43
VGG16 & SqueezeNet	0.60	0.79	0.78	0.81	0.64	0.93	0.48
VGG19 & GoogleNet	0.79	0.74	0.78	0.84	0.64	0.95	0.47
VGG19 & InceptionV3	0.78	0.79	0.76	0.88	0.69	0.97	0.43
VGG19 & ResNet50	0.76	0.81	0.72	0.88	0.66	0.93	0.55
VGG19 & ResNet101	0.72	0.79	0.74	0.88	0.66	***0.98***	0.41
VGG19 & InceptionResNetV2	0.78	0.84	0.79	0.88	0.64	0.95	0.47
VGG19 & SqueezeNet	0.60	0.78	0.76	0.86	0.57	0.95	0.52
GoogleNet & InceptionV3	0.88	**0.86**	0.74	0.79	0.62	0.90	0.47
GoogleNet & ResNet50	0.91	0.78	0.78	0.84	0.62	0.84	0.57
GoogleNet & ResNet101	0.88	0.78	**0.86**	0.79	0.62	0.90	0.55
GoogleNet & InceptionResNetV2	0.84	0.79	0.67	0.78	0.62	0.84	0.47
GoogleNet & SqueezeNet	0.47	0.57	0.79	0.83	0.59	0.86	0.53
InceptionV3 & ResNet50	**0.97**	0.81	0.79	0.83	**0.71**	0.90	0.50
InceptionV3 & ResNet101	0.88	0.83	0.74	0.81	0.66	0.86	0.50
InceptionV3 & InceptionResNetV2	0.84	0.78	0.76	0.84	0.66	0.84	0.57
InceptionV3 & SqueezeNet	0.57	0.62	0.78	0.88	0.66	0.93	0.53
ResNet50 & ResNet101	0.88	0.84	0.71	0.84	0.62	0.90	0.47
ResNet50 & InceptionResNetV2	0.93	0.83	0.76	0.84	0.60	0.91	0.41
ResNet50 & SqueezeNet	0.64	0.67	0.83	0.86	0.62	0.91	0.52
ResNet101 & InceptionResNetV2	0.84	0.79	0.79	0.86	0.67	0.84	0.45
ResNet101 & SqueezeNet	0.59	0.53	0.81	0.88	0.55	0.93	0.53
InceptionResNetV2 & SqueezeNet	0.62	0.59	0.81	0.90	0.57	0.91	0.48

The highest accuracy for each classifier is highlighted in bold font.

The highest overall accuracy is highlighted in bolditalic font.

The contributions of the two feature extractors are joined together to produce a more robust model with significantly better accuracy. This is easily noticed by boosting the best classification accuracy from 95% to 98% for the experiments of the single and double features, respectively. Of course, those best accuracies were produced using the SVM classifier.

The concatenated feature extractors that produced the best classification accuracy are VGG19 and ResNet101. We can still observe the contribution of ResNet architectures in producing the best results for each classifier. Out of the seven best accuracies in the seven classifiers, ResNet architectures were one of the two feature extractors used to produce the best six accuracies. Those classifiers are the SVM, DNN, Linear SVM, Discriminant, Naïve Bayes, and Tree classifiers, as can be seen from Table 5. This again supports the previous findings of ResNets as the best feature extractors.

SVM again shows good classification accuracies for each pair of the given feature extractors. Almost always, an Inception architecture was presented for every drop in the classification accuracy of the double features using SVM, as was noticed in the single-feature experiments. That emphasizes the fact that Inception architectures are bad feature extractors for radiology medical images. The worst accuracies reported using SVM were 84% and 86%, and they always involve an Inception architecture as one of its feature extractors. Finally, we again notice that the worst classifiers are gain Tree and Naïve Bayes classifiers with 71% and 59% best accuracies, respectively.

It is well shown in the results of the double-features experiments, that feature concatenation significantly improves the classification accuracy for each classifier. Except for the Tree and Naïve Bayes classifiers, where there was no significant improvement in the performance between single and double features using those two classifiers. That makes those two classifiers a bad choice for practitioners when dealing with radiology medical datasets.

### Discussion of the results

To summarize the results and outcomes represented earlier, we shortly discuss the effect of each of the used techniques in boosting classification performance. We show how this systematic exploration enabled us to achieve state-of-the-art results. Moreover, these results enabled us to understand the behavior of each classifier on medical datasets. They also enabled us, practitioners, and researchers to shorten the time required to find the best technique to use on new, similar datasets in the future.

Our systematic exploration of the used techniques on the LDR dataset enabled us to boost the previous state-of-the-art on this dataset that was achieved by^24^. The accuracy was improved from 91.38 to 98% using fine-tuning on the AlexNet pre-trained model. The fine-tuning accuracy was achieved by freezing the learned parameters on the first ten layers and increasing the learning factor on the newly added layers. This new state-of-the-art was also achieved using SVM on the concatenated features of VGG19 and ResNet101. However, we have also achieved other results better than the state-of-the-art with several single/double features mostly using SVM.

We did not test the feature Concatenation effect for fine-tuning experiments, i.e. ensemble fine-tuning. However, it is well noted that feature concatenation often had a good effect in enhancing the performance of classifiers if used with data augmentation. Hence, it is still recommended to investigate the effect of ensemble fine-tuning on these datasets to complement this work. Furthermore, SVM was mostly the best classifier on the extracted features, either used with single or double features. It is also important to reinforce the fact found by^28^, that ResNet architectures are usually more successful than any other architectures when used as feature extractors.

## Conclusion and future work

As a conclusion, this systematic exploration enabled us to achieve state-of-the-art results on the LDR dataset. It provides a comparative study that shows the effect of the different techniques discussed in this work, individually and combined. It is noticed from the discussed results, that fine-tuning transfer learning technique always produced the best classification accuracy results for radiology images. Adding rotation invariance to dataset images significantly helped boost the performance of every technique used in this study. Moreover, joining the outputs of different feature extractors has a significant performance improvement, especially when used with data augmentation.

It has been confirmed in this work, that ResNet architectures are almost often the best feature extractors for radiology medical image datasets. In this work, ResNets produced the best results when used as single or concatenated feature extractors. When working on the extracted features, SVM produces the best results. With the help of this systematic exploration, we were able to produce a state-of-the-art accuracy of 98% on the LDR dataset.

A good future direction is to perform ensemble fine-tuning of more than one pre-trained model at the same time, which requires powerful computational resources. We believe that it is worth experimenting given the success of this method in other domains. Furthermore, we will work on increasing the number of images in the LDR dataset by collaborating with healthcare providers and institutions. Expanding the dataset is a crucial step in enhancing the robustness and generalizability of our diagnosis system.

In addition, we plan to validate these findings on different types of medical images, i.e., Human Epithelial type 2 cells (HEp-2)^62^ microscopic medical image dataset that is captured using Indirect ImmunoFluorescence (IIF). This will show the generalization ability of the proposed system on a larger and more diverse dataset with different clinical settings, i.e., light microscopic imaging instead of MRI imaging. Finally, an ensemble fine-tuning approach should be considered as a future work. It is important to evaluate the effect of concatenating the last learnable layers of each of the two pre-trained models, or even more, to fine-tune them at the same time. That is worth investigating because fine-tuning produced the best results on our dataset. Since feature concatenation enhanced the performance of every model, it is necessary to evaluate the performance of ensemble fine-tuning techniques.

## Data Availability

The dataset analyzed during the current study is not publicly available because they are owned by the Jordan University of Science and Technology, but is available from the corresponding author upon reasonable request.
